# Modelling and Characterisation of Residual Stress of SiC-Ti_3_C_2_T_x_ MXene Composites Sintered via Spark Plasma Sintering Method

**DOI:** 10.3390/ma15031175

**Published:** 2022-02-03

**Authors:** Mateusz Petrus, Jarosław Woźniak, Marek Kostecki, Tomasz Cygan, Agnieszka Jastrzębska, Anita Rozmysłowska-Wojciechowska, Bogusława Adamczyk-Cieślak, Dorota Moszczyńska, Maksymilian Sienkiewicz, Piotr Marek, Arkadiusz P. Gertych, Mariusz Zdrojek, Andrzej Olszyna

**Affiliations:** 1 Faculty of Material Science and Engineering, Warsaw University of Technology, Woloska 141 St., 02-507 Warsaw, Poland; jaroslaw.wozniak@pw.edu.pl (J.W.); marek.kostecki@pw.edu.pl (M.K.); tomasz.cygan@pw.edu.pl (T.C.); agnieszka.jastrzebska@pw.edu.pl (A.J.); anita.wojciechowska.dokt@pw.edu.pl (A.R.-W.); boguslawa.cieslak@pw.edu.pl (B.A.-C.); dorota.moszczynska@pw.edu.pl (D.M.); andrzej.olszyna@pw.edu.pl (A.O.); 2 Institute of Aeronautics and Applied Mechanics, Warsaw University of Technology, Nowowiejska 24 St., 00-665 Warsaw, Poland; msienkiewicz@meil.pw.edu.pl (M.S.); piotr.marek@pw.edu.pl (P.M.); 3 Faculty of Physics, Warsaw University of Technology, Koszykowa 75 St., 00-662 Warsaw, Poland; Arkadiusz.Gertych@pw.edu.pl (A.P.G.); mariusz.zdrojek@pw.edu.pl (M.Z.)

**Keywords:** ceramic matrix composites (CMC), Ti_3_C_2_ MXene, mechanical properties, residual stress, FEM, numerical study, Raman spectroscopy

## Abstract

This article presents an attempt to determine the effect of the MXene phase addition and its decomposition during sintering with the use of the spark plasma sintering method on mechanical properties and residual stress of silicon carbide based composites. For this purpose, the unreinforced silicon carbide sinter and the silicon carbide composite with the addition of 2 wt.% of Ti_3_C_2_T_x_ were tested. The results showed a significant increase of fracture toughness and hardness for composite, respectively 36% and 13%. The numerical study involving this novel method of modelling shows the presence of a complex state of stress in the material, which is related to the anisotropic properties of graphitic carbon structures formed during sintering. An attempt to determine the actual values of residual stress in the tested materials using Raman spectroscopy was also made. These tests showed a good correlation with the constructed numerical model and confirmed the presence of a complex state of residual stress.

## 1. Introduction

Oxygen-free ceramics such as silicon carbide offer exceptional mechanical properties such as high hardness, high friction wear resistance, acid resistance, thermal resistance and good thermal conductivity [1,2]. Unfortunately, its low fracture toughness effectively limits its potential use [3]. One of the methods of improving the fracture toughness, while keeping its other properties unchanged, is the production of composites on its matrix [4].

In recent years, ceramic composites reinforced with two-dimensional crystals such as graphene family materials (GFM) have gained in importance [5]. As demonstrated in many studies on this subject, even a small addition of graphene flakes causes a significant increase in mechanical properties such as hardness, fracture toughness and performance properties (tribological or cutting) [6,7,8]. The increase in fracture toughness in these materials has been explained by the introduction of strengthening mechanisms into the matrix such as crack bridging, deflection or branching the propagating fracture by the flakes [9,10]. A new very promising family of two-dimensional crystals are the MXene phases, developed for the first time in 2011 [11]. Due to their unique properties, their geometry and the presence of functional groups on the surface, they are a promising material for use as reinforcement in composites based on the polymer and metal matrix [12,13,14]. However, introducing them into the ceramic matrix is, unfortunately, a difficult task mainly due to the relatively low thermal stability depending on the environmental conditions, which range between 300 °C and 1100 °C [15,16]. Nevertheless, there are works presenting an attempt to obtain ceramic/MXene composites. Guo et al., have obtained ZnO-MXene composites by using the CSP sintering method, which allows the use of a sintering temperature below the onset of MXene phase decomposition [17]. The works [18,19] also showed that despite the decomposition of the Ti_3_C_2_ phase during sintering, the decomposition products can modify the microstructure of the sinters, thus improving the mechanical properties of Al_2_O_3_ and Si_3_N_4_. In previous works [20,21] it was also shown that during the sintering of SiC-Ti_3_C_2_ composites via the spark plasma sintering method, the MXene phase decomposes with the formation of carbon graphite-like structures. These structures retain the geometry of the MXene flakes and therefore play a role as a reinforcing phase to enhance fracture toughness and hardness. As in the case of graphene-reinforced ceramic composites, the increase in fracture toughness was associated with the introduction of mechanisms such as delamination, branching, deflection and crack bridging into the matrix.

One important factor, apart from the microstructural factors that can have a significant impact on the mechanical properties, is the presence of internal stresses [22]. Depending on the size of the area they affect, they can be divided into stresses I, II and III types. The presence of internal stresses is particularly important in the case of ceramics subject to fracture toughness [23].Tensile residual stress leads to the formation of micro-cracks, which has a negative influence on fatigue life. On the other hand, comprehensive residual stress develops the strength and fracture toughness of materials [24,25,26,27]. Ceramic matrix composites are expected in the presence of tensile and compressive residual stresses due to the thermal expansion mismatch between the different phases and the thermal expansion anisotropy of different phases during the cooling step [28,29]. Due to their structures carbon structures such as graphene or graphite show an anisotropy of properties, including anisotropy of the thermal expansion coefficient [30,31,32]. During the heating and cooling of the composites reinforced with such crystals, fields of variable stress may form around it, which significantly affect the mechanical properties of such composites. The second factor that can significantly affect both the mechanical properties of ceramics and the presence of residual stresses is the sintering technique used. SPS is a fast sintering technique that achieves quick compaction results with minimal grain growth in a short sintering time. The increased sinterability of the powders subjected to SPS is mainly related to the activation of the surface of the particles and the increased diffusion rates in the contact zones caused by the applied pulsating current [33,34]. Due to the unique method of heat supply the use of this technique also allows the obtainment of a new generation of materials that cannot be produced by conventional sintering methods [35,36].

In this study, the influence of the MXene phase addition and the carbon structures’ formation during sintering on the presence of stresses in composites based on silicon carbide was analysed. MXene phases, as shown in previous studies [37], decompose during the sintering process to form highly defective carbon structures that play the role of a reinforcing phase. Due to the anisotropy of the thermal properties of graphite structures, the presence of internal stresses in the produced composites should be expected. For this purpose, the unreinforced silicon carbide sinter and the composite with the addition of 2 wt.% were subjected to stress analysis using Raman spectroscopy. Additionally, to support the experiment a novel finite element method [38] modelling technique has been developed.

## 2. Materials and Methods

### 2.1. Preparation of the Composites

A detailed description of the process of producing SiC-Ti_3_C_2_ composites was presented in the work [20]. In short, the MAX phase was obtained by using a self-propagating high-temperature synthesis process with the assist of a spark plasma sintering furnace (SPS). For this purpose, pure titanium powder (Goodfellow Cambridge Ltd., Cambridge, UK) with a chemical purity of 99.6% and the average particle size below 20 μm, pure aluminum powder with a chemical purity of 99.7% and synthetic graphite powder (Sigma-Aldrich, St. Louis, MO, USA) with a chemical purity of 99.9% and the average particle size below 20 μm were used. The molar ratio of Ti:Al:C = 3:1:1.9 was applied. The Ti_3_C_2_T_x_ MXene phase was obtained by etching the Ti_3_AlC_2_ MAX phase with a concentrated 48% hydrofluoric acid solution (no. 695068, Sigma-Aldrich, Taufkirchen, Germany). The ratio of the acid used in the process was 10 cm^3^ per 1 g of powder [39]. The composite was prepared with the powder metallurgy technique and sintered with the use of the SPS method. The powder mixture of SiC-2wt.% Ti_3_C_2_Tx was obtained via the powder metallurgy technique (stirring for 8 h using a planetary ball type mill). Propan-2-ol and alumina grinding balls were applied in this step. Composite was sintered with the use of the spark plasma sintering method. The parameters of the process were as follows: sintering temperature 1900 °C, heating and cooling rate 150 °C/min, 30 min dwell time, 50 MPa applied pressure and vacuum. As a reference sample, the silicon carbide sinter with the carbon and boron was produced.

### 2.2. Characterisation of the Composites

The density of the specimens was examined using the Archimedes method (PN-EN 1094-4:1998 Standard). Hardness was measured with the Vickers hardness tester (FV-700e, Future-Tech, Kawasaki, Japan) using the indentation method under the load of 49.05 N. The fracture toughness of the produced composite was determined by the Vickers indentation crack length method under the load of 49.05 N. The Niihara, Morena and Hasselman equation was used. Twenty hardness measurements and 12 crack length measurements were made for each sample. The phase composition of sinters was analysed with X-ray diffraction (XRD, D8 ADVANCE, Bruker Corporation, Billerica, MA, USA), using a CuKα radiation at a wavelength λ = 0.154056 nm. The parameters of the test were as follows: voltage 40 kV, current 40 mA, angular range 200°–100° and step 0.05°. The microstructure observations were performed on a scanning electron microscope (SEM Hitachi 5500, Tokyo, Japan).

### 2.3. Modelling of Residual Stress in Composites

**Figure 1:**
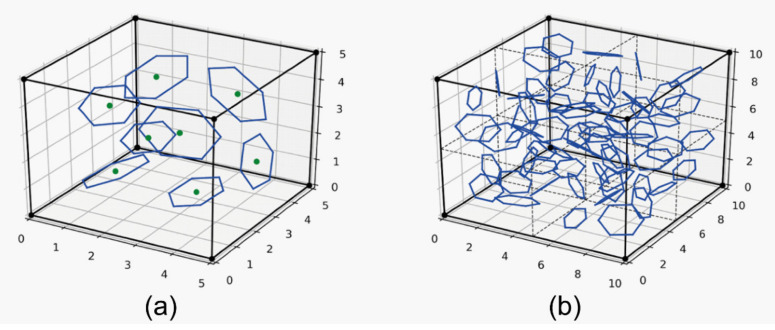
Geometrical models for hexagonal flakes in (**a**) a building block of dimension 5 μm × 5 μm × 5 μm with 8 flakes and (**b**) an exemplary cubic model consisting of 8 building blocks and 64 flakes.

The basic building block in analysis was a 5 μm × 5 μm × 5 μm cube containing 8 flakes, as shown in Figure 1a. The number of the flakes inside a block was calculated on the basis of the volume fraction reported from experiments. Each flake was modeled as a hexagonal as it better simulated the real shape of the MXene flakes [40,41]. The thickness of every flake was set to 0.5 micrometer.

The program developed for this research allows the analysis of cuboids consisting of any number of aforementioned building blocks, as shown in Figure 1b. After setting the size of the entire model, the appropriate number of flakes was generated inside in a uniform manner. Their sizes, positions and rotations were then randomly changed in order to render the chaotic distribution of a real sample. Since the flakes were assumed to have an orthotropic material properties, a local coordinate system was created for each of the flakes. Next, the geometrical data was transferred to the ANSYS [42] software to create a mesh and to perform the calculations.

In our case, the main focus was the stress state in the vicinity of a single flake, therefore only one block taken from the bigger model was sufficient for analysis. The materials used for the ceramic matrix and the reinforcing flake is shown in Table 1 and Table 2. The thermomechanical properties of the silicon carbide matrix were temperature dependent.

**Figure 2:**
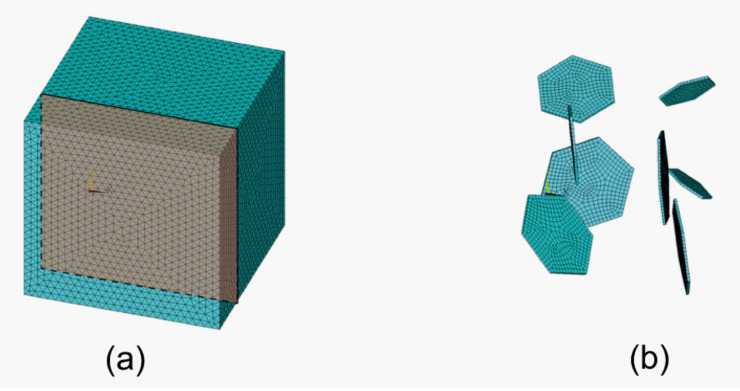
The finite element method models used in the analysis of (**a**) the entire model with a cross section used to show results and (**b**) the meshed flakes.

The entire model was meshed with the element SOLID186. The silicon carbide matrix was meshed with the 10-node tetrahedral elements. For the graphite flakes the 20-node hexahedral version of said element was chosen to ensure more accurate results. The resultant finite element model consists of 595,589 elements and 804,731 nodes (Figure 2). The flakes were assumed to be bonded with the matrix (i.e., no contact elements were added between them).

The boundary conditions were applied in a manner that allowed for the most accurate modelling of the manufacturing process. The analyzed model was only a small fragment of the entire element; therefore, proper deformation had to be considered that accounted for the rest of the material; therefore, symmetry boundary conditions were applied to the back surfaces (x = 0, y = 0, z = 0) of the model. On two surfaces (x = 5, y = 5) the node coupling was utilized to ensure uniform deformation. Lastly, the front surface (z = 5) had no boundary conditions applied to model the free surface of the specimen. It was assumed that the flakes of the reinforcing phase and the ceramic matrix showed perfect adhesion at the end of the sintering process and that this state was stress-free. Later, in the cooling phase (from 1900 °C to 2000 °C) only the linear behavior of the materials was taken into account. Unlike the others publications [41,43], where MXene flakes were modelled as circles, the presented modelling technique concerned petals modelled as polygons (the shape is a parameter), which is closer to the real shape. The modelling technique developed during the research allowed the analysis of the impact of various parameters: the spatial distribution of flakes, their sizes and shape and the angles of inclination or their number.

### 2.4. Residual Stress Analysis

Raman measurements were performed using a spectrometer in a backscattering configuration with a motorized XYZ stage that offered 100 nm resolution (inVia Qontor, Renishaw, Warsaw, Poland). All spectra were collected using a 100× objective with the numerical aperture of 0.85, circularly polarized light, 532 nm laser, 4 mW of laser power and 1800 lines/mm grating. On each sample, mapping measurements with an area 100 µm × 100 µm and a step equal to 1 µm in x and y direction were performed. A Lorentz function with a linear background was fitted to each Raman mode to extract the peak position from our Raman spectra. In the case of a split TO mode three Lorentzian functions were fitted in order to obtain good agreement between experimental data.

## 3. Results

**Figure 3:**
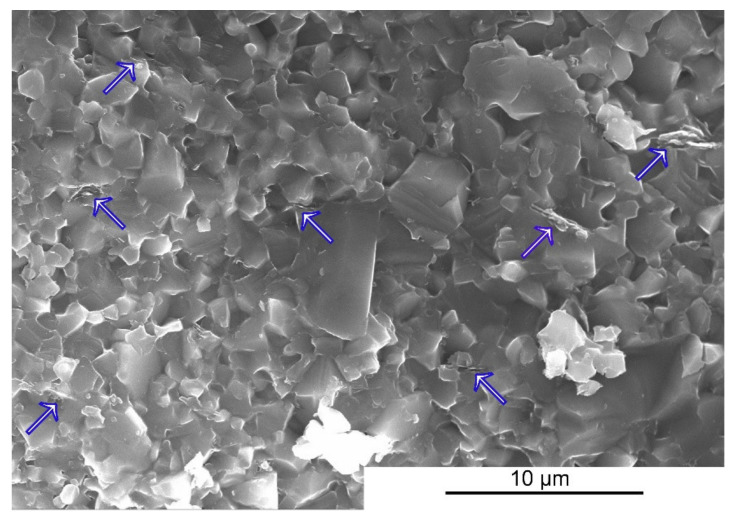
The fracture surface of the SiC+2wt.% of Ti_3_C_2_ composite.

The physical and mechanical properties of the sinters are presented in Table 3. The addition of 2wt.% of the Ti_3_C_2_ MXene phase caused an increase in hardness by over 10% and an increase in fracture toughness by over 36%. As shown in previous works [21], during the sintering of composites the MXene flakes decompose with the formation of graphite-like disordered carbon flake structures that play the role of reinforcing the phase and are responsible for the observed changes in the mechanical properties. The increase in hardness can be explained by a decrease in the average grain size of the carbide matrix. These flakes are located at the grain boundaries (Figure 3), blocking their growth during sintering by nearly 30%. The increase in fracture toughness, as in the case of the ceramic composites reinforced with materials from the graphene family, is caused partly by the introduction of reinforcing mechanisms into the material such as delamination, crack bridging or the branching on the flakes of the reinforcing phase located at the grain boundaries.

**Figure 4:**
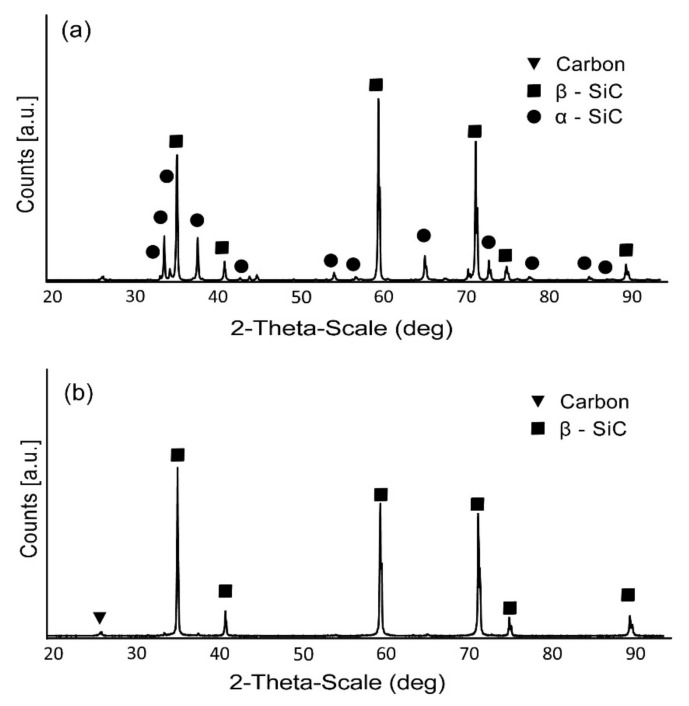
The XRD results of (**a**) SiC0 and (**b**) SiC2 samples.

Figure 4 shows the analysis results of the phase composition of the produced sinters. The pure silicon carbide sinter exhibits a multi-phase structure. Apart from the β-SiC phase, the presence of the α-SiC phase and a trace amount of carbon were also found. The SiC2 sample only confirmed the presence of the β-SiC phase and, similarly to the reference sinter, a small amount of carbon.

**Figure 5:**
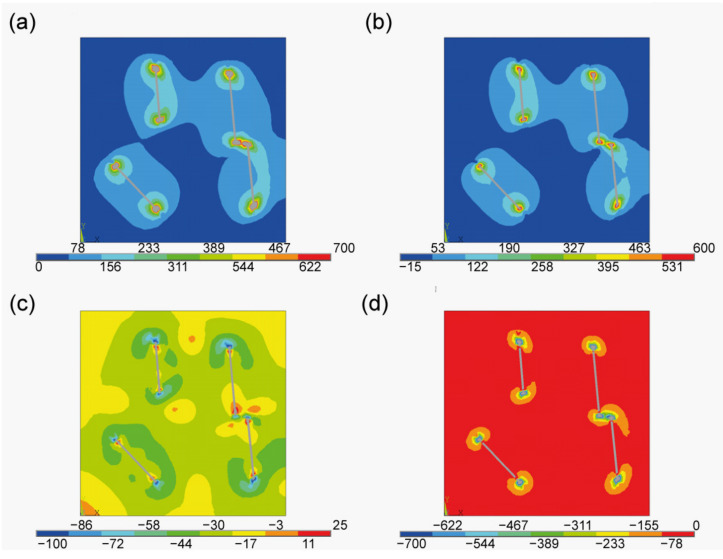
Stress state at the cross section. (**a**) Equivalent stress, (**b**) principal stress S1, (**c**) principal stress S2, (**d**) principal stress S3 (units in MPa).

**Figure 6:**
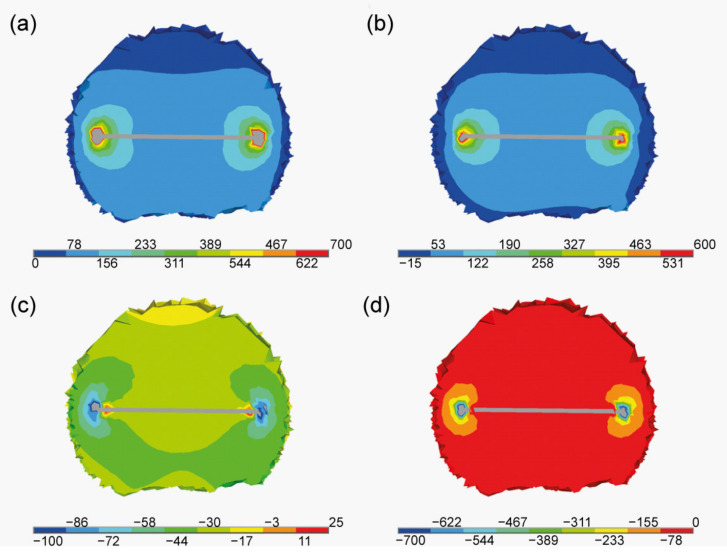
Stress state in the proximity of one of the flakes. (**a**) Equivalent stress, (**b**) principal stress S1, (**c**) principal stress S2, (**d**) principal stress S3 (units in MPa).

The main point of interest for our study was to determine the stress state around the given flake and to check its repeatability for other flakes present in the given material. Special attention was given to the principal stresses, as they can determine whether the flake is under tension or compression. Principal stresses S1, S2 and S3 obtained in the cross section are shown in Figure 5 and Figure 6. Figure 5 presents the general distribution for the entire cube; whereas, Figure 6 shows the detailed distribution in the vicinity of the sample flake. Besides the principal stress results, the distribution of the equivalent stresses was presented.

**Figure 7:**
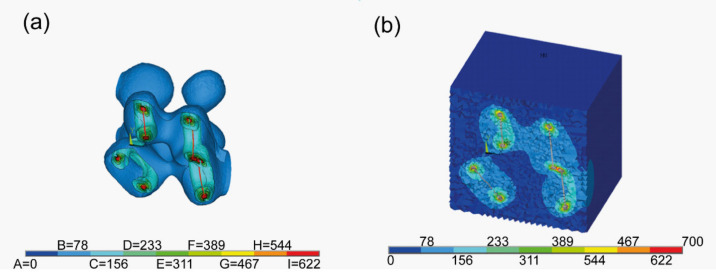
Von Mises equivalent stresses at the cross section. (**a**) Isosurfaces and (**b**) distribution (units in MPa).

The first principal stress S1 is connected to the in-plane flake direction. As seen in Figure 6b, the concentration occurs at the borders of the flakes. This mean that the flakes are being stretched in their plane. This effect was expected [43,44] as it connected to the mismatch of the thermal expansion coefficients between the flakes and the matrix. The next principal stresses are related to the tangent S2 and the perpendicular S3 directions (in regards to the flake). In both cases those values are negative in the vicinity and at the border of the flakes. This implies the occurrence of the compressive state. In both cases the compressive stress is strongly localised on the flake’s border. In Figure 7 the equivalent stress is presented. Their distribution proves that the disturbance is localised near to the flake. This behaviour can be clearly seen in Figure 7a, where values are presented as isosurfaces, i.e., surfaces of constant stress value.

**Figure 8:**
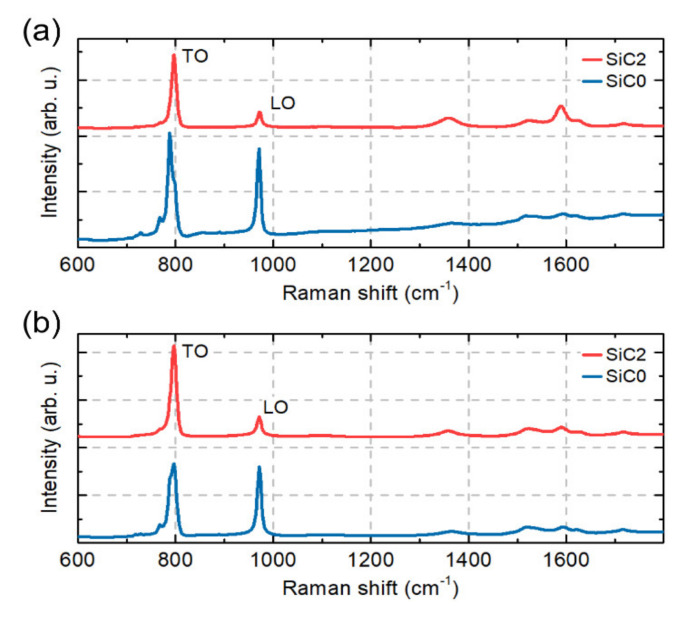
Average Raman spectra for SiC0 and SiC2 samples collected (**a**) on the polished surface and (**b**) on the fracture surface.

To compare the macro properties of our samples, the collected Raman maps were averaged. The spectra obtained are shown in Figure 8. In the SiC2 sample, typical Raman modes TO and LO corresponding to the β-SiC phase are visible. In the SiC0 samples it can be seen that the TO Raman mode is divided into three modes. In the case of the SiC modes, no difference was noticed between the measurements on the polished and the fracture surfaces. However, it can be seen that the measurements of the polished surface show an increased carbon content in the SiC2 sample due to the increased intensity of the Raman carbon modes around 1580 cm^−1^ and 1350 cm^−1^.

**Figure 9:**
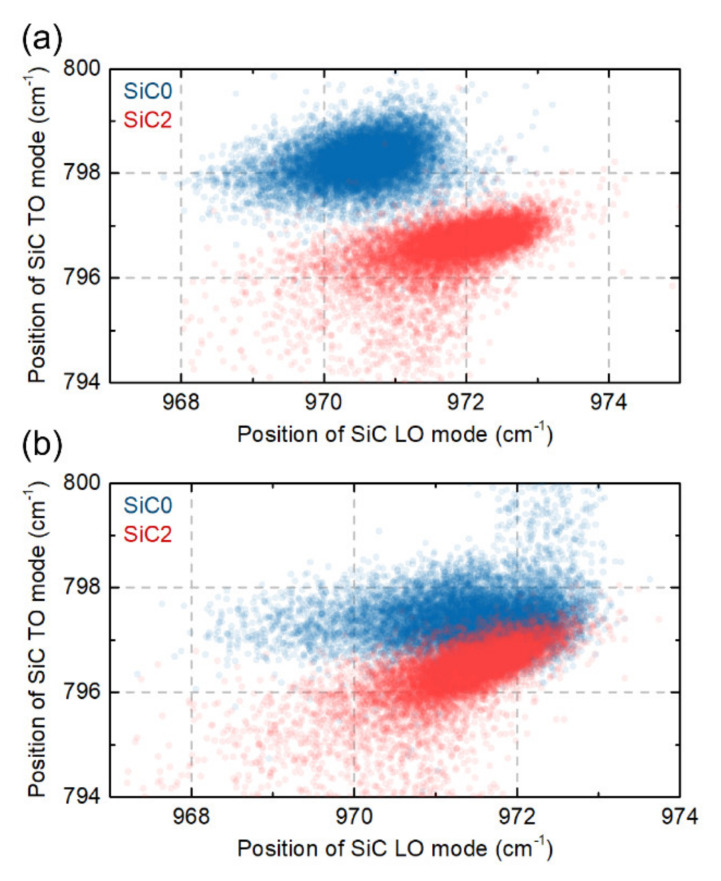
SiC TO mode position as a function of SiC LO mode position measure for SiC0 and SiC2 samples on (**a**) the polished surface and (**b**) the fracture surface.

The change in the Raman peak position of LO and TO SiC modes are often used to measure strain in SiC samples [45,46,47]. To see the difference in the position of the LO and TO modes between our samples, the SiC TO mode position as a function of the SiC LO mode position for 10,000 points collected in our mapping measurements is shown in Figure 9. The statistical results show that the observed changes in the position of the TO and LO mode peaks between the SiC0 and SiC2 samples for the polished surface and the fracture surface. It can be seen that this change is more pronounced in the maps made on a polished surface.

To show the spatial distribution of the peak position and strain the SiC LO peak was analysed, because it retains its shape in all of our samples, thus minimising the uncertainty resulting from the fitting procedure. Since the SiC0 and SiC2 samples have partly different phases, the stress distribution is calculated as follows: For each map the mean peak position was first calculated, then for each point on the map the difference between the mean peak position (*ω*_average_) and peak position in point (*ω*_point_) was measured. Finally, the conversion factor from the literature was used to convert the difference in peak position to stress level $\frac{d\omega }{d\sigma }=4.27\;{\mathrm{cm}}^{-1}/\mathrm{GPa}$ [48,49]. This operation can be summarized with the following formula:

$$\sigma =\frac{\left(\omega_{\mathrm{point}}-\omega_{\mathrm{average}}\right)}{\frac{d\omega }{d\sigma }}$$

Here, note that to extract spatial distributions it was assumed that there was only one phase—β-SiC—in all samples, and that the difference in peak position came mainly from stress.

## 4. Discussion

As was shown before [20,21], during sintering of the SiC-Ti_3_C_2_ MXene composites the decomposition of MXene flakes takes place with the formation of graphite-like carbon structures that act as a reinforcing phase, which increases both hardness and fracture toughness. The proposed mechanism of the formation of disordered graphitic carbon structure assumes that during the sintering the silica coating of the SiC powder evaporates into the area between grains. In higher temperatures gaseous SiC is then also created as a result of thermal decomposition. Moreover, the MXene phases tend to lose oxygen-containing functional groups (–OH, =O). As a result, the factors responsible for the formation of disordered graphitic carbon structure can be found in the close vicinity of the MXene phases during the sintering process [50]. This phenomenon is possible through the use of the SPS sintering technology, which (due to the method of heat supply) removes oxides from the grain surface, releases functional groups from the MXene surface and performs the sintering process in an extremely short time. This allows the abovementioned mechanism to occur and limits further degradation of the phases of forming carbon structures. The use of SPS sintering technology also made it possible to obtain a single-phase structure in the case of the produced composite. The β-SiC phase is unstable in a wide temperature range; therefore, during sintering with conventional sintering methods the β-SiC → α-SiC phase transformation occurs [51]. In the case of the SPS sintering technology, this phenomenon is significantly reduced due to the short time of the entire process. A small amount of the α-SiC phase was confirmed in the case of the unreinforced sinter, which may indicate that the presence of MXene phases may additionally inhibit the phase transformation of silicon carbide, similar to the case of composites with the addition of graphene [52].

**Figure 10:**
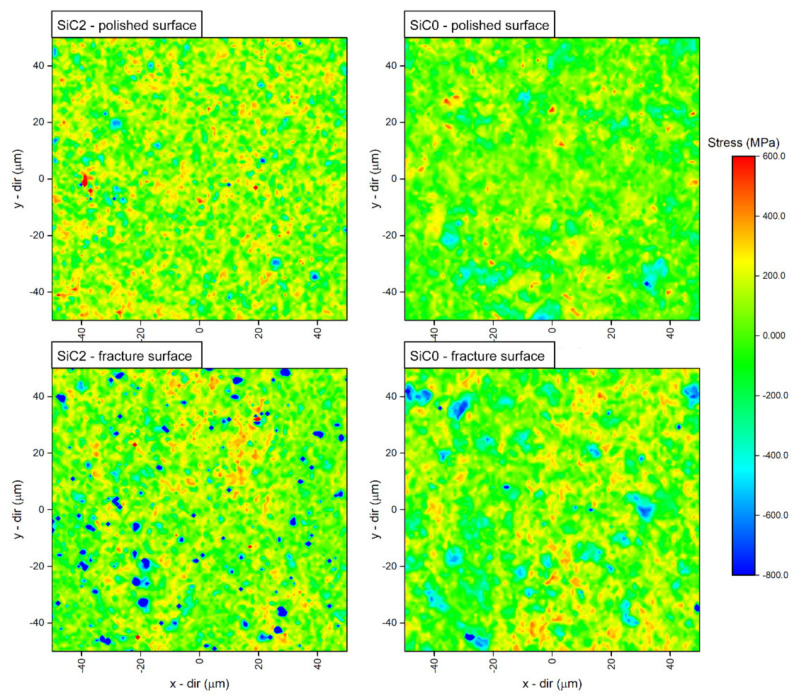
Spatial distribution (mapping) of the stress level for SiC0 and SiC2 samples extracted from the polished sample and fracture surfaces.

Graphite and graphite-like carbon (due to its crystal structure) show a strong anisotropy of mechanical and thermal properties, including the anisotropy of the thermal expansion coefficient [53]. The mismatch of the expansion coefficients in the case of ceramic composites, in combination with the above-mentioned anisotropy of one of the phases, can significantly affect the level and distribution of residual stresses [54]. This assumption was confirmed in the conducted numerical study, the results of which show the qualitative state of stress in the vicinity of the flakes remaining from the Ti_3_C_2_ MXene phase. It indicates a strong localization of compressive stresses around the edge of the flake. According to the analysis, they can reach values close to 700 MPa. However, it should be noted that the model is based on the basic linear behaviour of the material during cooling. It accepts many simplifications such as: no phase transformation, perfect interface between the flakes and the matrix, no grain growth or no formation of agglomerates of the reinforcing phase. Due to all of the above assumptions, the macroscale model itself may be burdened with many errors and may not reflect the actual state of stresses in the material. However, on the micro scale, i.e., the area directly around the flakes of the reinforcing phase, some of these simplifications have little effect on the obtained results. Therefore, it can be concluded that the constructed model describes the distribution of residual stresses on the microscale with greater accuracy, which is indicated by the high correlation of the obtained results. The validation of the model with a much larger number of experimental tests in the microscale, including the remaining elements of the microstructure in the model contributing to the overall stress state of the material (pores and interfacial boundaries), will allow for the building of more effective tools for the accurate description of real materials in the macroscale. It should also be emphasized that carrying out such tests is related to the appropriate preparation of samples such as cutting or polishing. Taking into account the differences in the stress distribution between the polished surface and the fracture surface (Figure 10), it can be assumed that these tests could also be burdened with a large error, which makes the validation of such a model much more difficult.

The phase composition analysis of obtained composites showed that there is little α-SiC phase in the SiC0 sample. The SiC2 composite in turn is characterised by a single-phase structure. These results were confirmed in the analysis of an averaged Raman spectra. The observed Raman mode splits into three modes for the SiC0 sample, suggesting the appearance of the α-SiC phase(e.g., 6H and 15R) [47,55,56]. The Raman maps obtained for the SiC2 sample are also characterised by modes originating from the carbon structures, which confirms the thesis concerning the decomposition of MXene phases during the sintering process. The observed differences in the position of the LO mode between the SiC2 and SiC0 samples also suggest that the sample is heterogeneous in terms of stress distribution. The maps of stress distribution on the polished surface and fracture surfaces obtained on this basis confirm these assumptions. However, it should be emphasized that polishing the surface can significantly affect the state of internal stress [57], and the diamond suspensions of various sizes used in the polishing process may change the nature of stresses at a depth of up to several hundred nanometres [58,59]. This can significantly distort the information regarding the stresses that remain on the polished surface. However, it can be concluded that in the case of the produced materials the polished surfaces are characterised by the presence of tensile stresses.

More precise information can be provided by the analysis of the state of stresses on the fracture surface. It should also be noted that the comparison of the obtained maps is difficult due to the presence of the α-SiC phase in the SiC0. The ranges of the peak position changes related to the identification of the α-SiC phase are of the order of −2 cm^−1^ [47], while the changes of the peak position related to the presence of stresses are of the order of −4 cm^−1^/GPa [46,48]. As a result, if the sample is not single-phase the source of the observed position changes cannot be clearly determined. In the case of the SiC2 sample, in which the presence of the α-SiC was not observed, numerous areas characterised by compressive stresses were found. These areas are definitely present more often than in the case of the SiC0 sample. Their distribution corresponds to the results of the microscopic observations and the distribution of graphitic carbon flakes. Their shape and determined values reaching −700 MPa are confirmed in the performed numerical study. As mentioned earlier, in the case of the SiC0 sample the observed areas showing the presence of compressive stress may be connected with the presence of α-SiC phase grains; thus, it can be concluded that the presence of carbon flakes introduces a complex state of stresses into the ceramic matrix. According to the literature, compressive stress fields in such materials can significantly improve the fracture toughness. The fracture propagated in the area of such stresses will be effectively closed and deflected [44,60], which in combination with the mechanisms that increase the fracture toughness (such as delamination, bridging or deflection) observed in such materials will effectively increase the fracture toughness.

## 5. Conclusions

The conducted tests allow for the conclusion that the introduction of MXene phases into the ceramic matrix can effectively increase the hardness and fracture toughness. The analysis of the internal residual stresses revealed the presence of a complex state of stresses in the material, which is related to the anisotropic properties of graphitic carbon structures formed during sintering. The research was supported by a numerical study involving the novel method of modelling, which shows good correlation with experimental results in regard to the distribution of the stresses. The measurement of residual stresses in ceramic composites is extremely complex especially for materials such as silicon carbide, where the potential phase change can influence the obtained results. However, this problem is extremely important from the point of view of designing ceramic composites, due to their significant influence on the mechanical properties. The combination of numerical methods and real measurements is a very useful tool to better understand the effects of the reinforcing phase on internal residual stress, and thus a full description of changes in mechanical properties and their causes.

## Figures and Tables

**Table 1:** Thermomechanical properties used for the silicon carbide.

Properties	Symbol	Temperature		
		20 °C	1900 °C	Unit
Young’s Modulus	*E*	475	415	GPa
Poisson’s Ratio	*ν*	0.16	0.18	-
Conductivity	*k*	120	20	W/(m·K)
Specific Heat	*C_p_*	0.829	1.4	J (mol·K)
Thermal Expansion Coefficient	*α*	4.4 × 10^−6^	4.9 × 10^−6^	1/K

**Table 2:** Orthotropic thermomechanical properties used for the graphite material.

Properties of Graphite								
*E_x_*	50	Gpa	*G_xy_*	29,762	Gpa	*α_x_*	0.000025	1/K
*E_y_*	50	GPa	*G_yz_*	28,274	GPa	*α_y_*	0.000025	1/K
*E_z_*	475	GPa	*G_xz_*	28,274	GPa	*α_z_*	0.000002	1/K
*ν_xy_*	0.16	-	*k_xy_*	398	W/(m·K)	*C_p_*	0.829	J (mol·K)
*ν_yz_*	0.16	-	*k_yz_*	398	W/(m·K)			
*ν_xz_*	0.16	-	*k_xz_*	2.2	W/(m·K)			

**Table 3:** Properties of obtained sinters.

Material	Designation	Relative Density [%]	Average Grain Size [µm]	Hardness [GPa]	VIF [MPa·m^0.5^]
SiC	SiC0	98.4 ± 0.15	0.98 ± 0.04	20.7 ± 0.50	3.1 ± 0.22
SiC+2wt.% Ti_3_C_2_	SiC2	98.4 ± 0.25	0.71 ± 0.05	23.0 ± 0.67	4.22 ± 0.30

## Data Availability

The data presented in this study are available on request from the corresponding author.
